# Four new species of the trapdoor spider genus *Conothele* Thorell, 1878 from Mainland China and Laos (Araneae, Ctenizidae)

**DOI:** 10.3897/zookeys.643.10543

**Published:** 2017-01-05

**Authors:** Xin Xu, Chen Xu, Fengxiang Liu, Zengtao Zhang, Daiqin Li

**Affiliations:** 1 College of Life Sciences, Hunan Normal University, Changsha, Hunan, China; 2 Centre for Behavioural Ecology and Evolution (CBEE), College of Life Sciences, Hubei University, Wuhan, Hubei, China; 3 Department of Biological Sciences, National University of Singapore, 14 Science Drive 4, Singapore 117543

**Keywords:** Araneae, China, Conothele, Ctenizidae, Laos, taxonomy

## Abstract

Here for the first time the presence of the trapdoor spider genus *Conothele* Thorell, 1878 (Araneae: Ctenizidae) is reported from mainland China and Laos. Four *Conothele* species collected from the regions are described as new to science, based on the female genital morphology: *Conothele baiyunensis* Xu, Xu & Liu, **sp. n.** (Guangdong Province), *Conothele daxinensis* Xu, Xu & Li, **sp. n.** (Guangxi Province), *Conothele sidiechongensis* Xu, Xu & Liu, **sp. n.** (Yunnan Province, China and Vietnam), *Conothele yundingensis* Xu, Xu & Li, **sp. n.** (Yunnan Province).

## Introduction

The family Ctenizidae belongs to the suborder Opisthothelae and the infraorder Mygalomorphae. Ctenizids are widely distributed, and mainly found across Asia (China, India, Japan, Laos, Myanmar, Sumatra, Thailand, Vietnam), the Americas (North and South America), the Mediterranean, South Africa and Australia (World Spider Catalog 2016). Ctenizidae is a dispersal-limited, ground-dwelling lineage, members of which usually build underground silk lined burrows opening to the surface with a trapdoor. The trapdoor is sometimes covered with a layer of leaf litter or moss, which blends well with the surrounding environment, thus making it difficult to spot in the field (Gertsch and Wallace 1936; Hunt 1976; Platnick and Gertsch 1976; Bond and Coyle 1995).

Ctenizids were traditionally divided into two subfamilies based on morphological features, Ctenizinae and Ummidiinae (Raven 1985; Ortiz 2007). Ctenizinae includes six genera, *Bothriocyrtum* Simon, 1891, *Cteniza* Latreille, 1829, *Cyclocosmia* Ausserer, 1871, *Cyrtocarenum* Ausserer, 1871, *Latouchia* Pocock, 1901, and *Stasimopus* Simon, 1892. However, this grouping is not supported by any identified synapomorphies (Raven 1985). Ummidiinae contains three genera, *Conothele* Thorell, 1878, *Hebestatis* Simon, 1903, and *Ummidia* Thorell, 1875 and it is diagnosed by the presence of a saddle depression on the tibia III that may serve as an anchor in the burrow (Gertsch 1979; Coyle 1981, 1985). Recently Decae (2010) removed *Hebestatis* from this subfamily based on some distinct morphological characteristics, including the presence of lateral sternal sigilla, a less pronounced and glabrous dorsal saddle on the tibia III, an absence of curvy spines, a lack of tarsal clavate trichobothria, and the absence of centrally sclerotized spermathecae, which are not present in the other genera of Ummidiinae. Therefore, the taxonomic position of *Hebestatis* remains uncertain (but see Garrison et al. 2016). The phylogenetic structure of the family Ctenizidae is also contentious. Recent phylogenetic studies have recovered neither the monophyly of Ctenizidae nor of Ctenizinae (Hedin and Bond 2006; Ayoub et al. 2007; Bond et al. 2012; Opatava et al. 2013), even though Garrison et al. (2016) recovered *Cyclocosmia* and *Hebestatis* as monophyletic. Most importantly, *Cteniza*, the type genus of the family, which is consistently supported as sister to the other Mediterranean genus *Cyrtocarenum*, is never recovered as closely related to any of the remaining genera of the family (Raven 1985; Decae 1996; Opatova 2013). Similarly, the genus *Stasimopus* never clusters with any other Ctenizidae genus (Hedin and Bond 2006).

The genus *Conothele* is represented by 20 species distributed in the Oriental and Australian regions. The taxonomic status of *Conothele* has been a matter of debate. The genus is closely related to *Ummidia*, from which it only differs by characters of uncertain generic significance (Main 1957; Siliwal et al. 2009; Opatova et al. 2013), which has led some authors to consider *Conothele* as a junior synonym. However, the two genera show some differences in distribution and burrow architecture. Unlike *Conothele*, *Ummidia* is restricted to the New World and the Mediterranean region (Main 1957; Bond et al. 2012; Siliwal et al. 2015; World Spider Catalog 2016). In addition to the different geographical distributions, the burrowing habits of both genera are also entirely different: *Conothele* constructs a short superficial burrow (arboreal or ground) parallel to the ground surface, while *Ummidia* digs a deeper and longer burrow (Main 1957; Haupt 2006; Siliwal et al. 2009). To date, only eight *Conothele* species have been described from Asia, i.e., *Conothele birmanica* Thorell, 1887 (juvenile) from Myanmar, *Conothele cambridgei* Thorell, 1890 (juvenile) from Indonesia (Sumatra), *Conothele fragaria* (Dönitz, 1887) (♂♀) from Japan, *Conothele taiwanensis* (Tso, Haupt & Zhu, 2003) (♂♀) from Taiwan, *Conothele giganticus* Siliwal & Raven, 2015 (♀), *Conothele khunthokhanbi* Kananbala, Bhubaneshwari & Siliwal, 2015 (♀), *Conothele vali* Siliwal et al., 2009 (♀) and *Conothele varvarti* Siliwal et al., 2009 (♀) from India.

In this study, four new *Conothele* species are diagnosed and described based on the morphology of female specimens collected from mainland China and Laos, where the genus had not been reported before. Although, ideally both male and female characters should be included in the description of new species, in trap-door spiders obtaining males is extremely difficult and indeed we were unable to obtain male *Conothele* specimens in this study. Direct collection by searching and digging burrows primarily results in either females or immature specimens. Males are short lived and leave the burrow immediately after the adult moult to search for females (Haupt and Shimojana 2001; Haupt 2003). Therefore, collecting males is only possible at certain times of the year, and thus is not feasible during all collection trips.

## Materials and methods

Specimens were examined under an Olympus SZX16 stereomicroscope, and photographed using an Olympus BX51 compound microscope. Genitalia were cleaned in boiling KOH for a few minutes to dissolve soft tissues. All the specimens were deposited at the Centre for Behavioural Ecology and Evolution (CBEE), College of Life Sciences, Hubei University, Wuhan, China. All measurements are in millimetres. Leg and palp measurements are given in the following order: total length (femur + patella + tibia + metatarsus + tarsus).

Abbreviations used are:

ALE
 anterior lateral eye 


AME
 anterior median eye 


PLE
 posterior lateral eye 


PME
 posterior median eye 


MOA
 median ocular area 


PMS
 posterior median spinneret 


PLS
 posterior lateral spinneret 


## Taxonomy

### Genus Conothele

Taxon classificationAnimaliaAraneaeCtenizidae

Thorell, 1878

#### Type.

*Conothele malayana* (Doleschall, 1859): 5, pl. 5, figure 8 (described female).

#### Diagnosis.

The genus *Conothele* can be distinguished from the genus *Ummidia* by their burrowing habits, the former constructs a short, parallel to the surface of ground, superficial burrow, whereas the latter digs a several centimeters long burrow in the soil (Haupt 2006). Moreover, *Conothele* distributes in the Oriental and Australian regions, whereas *Ummidia* is only found from New World and the Mediterranean region (Haupt 2006; Siliwal et al. 2009; World Spider Catalog 2016).

### Conothele baiyunensis

Taxon classificationAnimaliaAraneaeCtenizidae

Xu, Xu & Liu sp. n.

http://zoobank.org/EBF1B2F7-6A80-4A4D-8FC6-4848E9195F81

Fig. 1

#### Holotype.

Female (XUC-2014-062), Mt. Baiyun, Guangzhou City, Guangdong Province, China, 23.294°N, 113.484°E, 20 June 2014, collected by F.X. Liu, C. Xu and Z.T. Zhang. No male found.

#### Etymology.

‘Baiyun’ refers to the type locality of this species.

#### Diagnosis.

**Figure 1.**
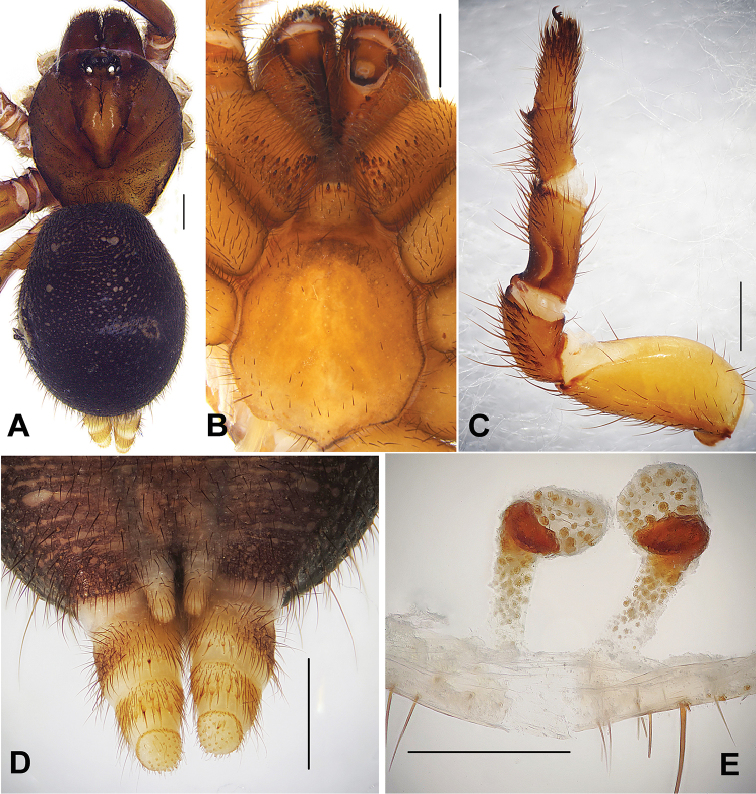
**A** General somatic morphology of *Conothele baiyunensis* Xu, Xu & Liu, sp. n. (female, XUC-2014-062) **B** chelicerae, labium, coxae of palp and sternum, ventral view **C** left leg III, prolateral view **D** spinnerets, ventral view **E** female genitalia, dorsal view. Scale bars: **A–D** 1 mm, **E** 0.5 mm.

Female of *Conothele baiyunensis* sp. n. can be distinguished from other species of *Conothele* by the slightly globular lobes of spermathecae in the terminal part; stalks with sclerotized and inward-bent distal part; stalk terminal parts relatively short, simple and direct (Fig. 1E).

#### Description.

Total length, including chelicerae, 12.50; carapace 4.80 long, 4.40 wide; opisthosoma 6.70 long, 5.20 wide. Carapace black brown, glabrous, with a few slender setae on eye tubercle and its back (Fig. 1A). Caput arched. Fovea deep and darker, strongly procurved and U-shaped (Fig. 1A). Eye tubercle low and black on its margin. Eight eyes in two rows, with the anterior eye row slightly procurved and posterior row straight from above (Fig. 1A); eye group 0.70 long, 1.25 wide; ALE-AME 0.20, AME-AME 0.10, PLE-PME 0.05, PME-PME 0.45; MOA 0.55 long, front width 0.40, back width 0.95; ALE: AME: PLE: PME (0.30: 0.15: 0.20: 0.25). Clypeus width 0.30. Chelicerae black; inner margin with 4 teeth, outer margin with 8 teeth. Labium, coxae of palp (maxillae) and sternum yellow brown (Fig. 1B). Labium 0.70 long, 0.80 wide, with 3 conspicuous cuspules (one absent). Coxae of palp (maxillae) 1.65 long, 1.10 wide, with about 21 conspicuous cuspules ventrally. Sternum 3.00 long, 2.40 wide, with large, irregularly shaped sigilla in the centre (Fig. 1B).

Legs black brown, light-coloured ventrally, with long and short brown sparse setae, short thorn-like and normal spines. Tibia III with a saddle-like depression dorsally on the basal part (Fig. 1C). Metatarsus III with three prolateral spines. Femur III thickest. Scopulae and claw tufts absent on trasi of all palp and legs. Palpal claw with a single branched tooth; legs each with three tarsal claws, paired claws with two denticles. Leg formula: 4123. Measurements of palp and legs: palp 7.35 (2.80 + 1.25 + 1.75 + 1.55), leg I 8.30 (3.10 + 1.55 + 1.75 + 0.95 + 0.95), leg II 7.50 (2.50 + 1.25 + 1.75 + 1.05 + 0.95), leg III 7.35 (2.50 + 0.90 + 1.65 + 1.10 + 1.20), leg IV 9.70 (3.25 + 1.25 + 2.00 + 1.90 + 1.30).

Opisthosoma black, scattered with thick and slender black setae. Spinnerets brownish yellow, PMS one-segmented, 0.60 long, PMS-PMS 0.10; PLS three-segmented, 1.85 long, covered with brown spines, apical segment dome-shape (Fig. 1D). Genitalia with paired slightly globular lobes of spermathecae in the terminal part, each stalk slender, long, at the distal part is sclerotized and bent, yet the bending is relatively short and looks more simple and direct (Fig. 1E).

#### Distribution.

Guangdong Province (Mt. Baiyun, Guangzhou), China.

### Conothele daxinensis

Taxon classificationAnimaliaAraneaeCtenizidae

Xu, Xu & Li sp. n.

http://zoobank.org/2D68220C-B6AA-4C0D-86B6-D796DB366FD4

Fig. 2

#### Holotype.

Female (XUC-2014-002+), Minghua Village, Daxin Town, Chongzuo City, Guangxi Province, China, 23.320°N, 107.728°E, 22 December 2014, collected by D. Li, F.X. Liu and X. Xu. No male found.

#### Etymology.

‘Daxin’ refers to the type locality of this species.

#### Diagnosis.

**Figure 2.**
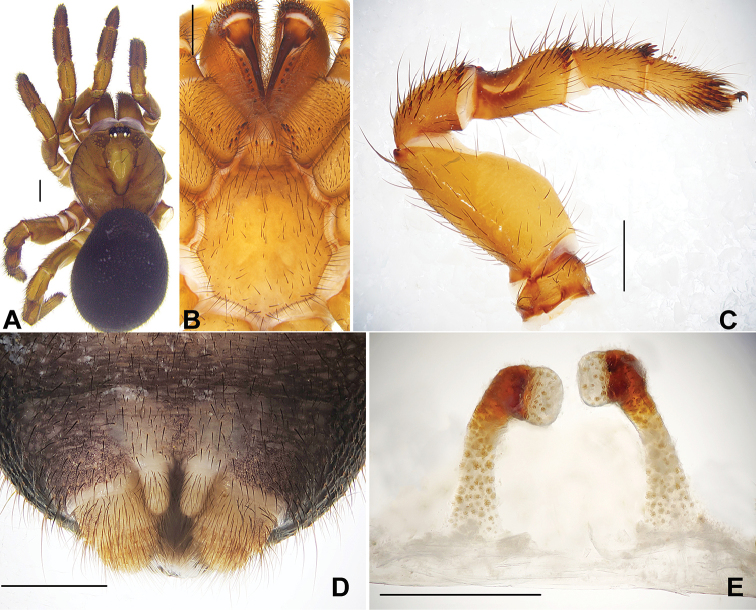
**A** General somatic morphology of *Conothele daxinensis* Xu, Xu & Li, sp. n. (female, XUC-2014-002+) **B** chelicerae, labium, coxae of palp and sternum, ventral view **C** left leg III, prolateral view **D** spinnerets, ventral view **E** female genitalia, dorsal view. Scale bars: **A–D** 1 mm, **E** 0.5 mm.

Female of *Conothele daxinensis* sp. n. differs from other species of *Conothele* by the spermathecae with face to face, bowl-shape lobes (Fig. 2E); each stalk slender, long, slightly broader at base, distally sclerotized and incurved (ca. 110°) (Fig. 2E).

#### Description.

Total length, including chelicerae, 10.40; carapace 4.00 long, 4.60 wide; opisthosoma 5.60 long, 4.90 wide. Carapace light brown, glabrous, with 3 slender setae on eye tubercle and 4 on its back (Fig. 2A). Caput arched. Eye tubercle black brown. Fovea deep, strongly procurved and U-shaped (Fig. 2A). Eight eyes in two rows, with both the anterior and posterior rows straight from above (Fig. 2A); eye group 0.60 long, 1.10 wide; ALE-AME 0.10, AME-AME 0.05, PLE-PME 0.04, PME-PME 0.30; MOA 0.55 long, front width 0.45, back width 0.96; ALE: AME: PLE: PME (0.30: 0.20: 0.13: 0.33). Clypeus width 0.25. Chelicerae black, inner margin with 4 teeth, outer margin with 9 teeth. Labium, coxae of palp (maxillae) and sternum light brown (Fig. 2B). Labium 0.55 long, 0.85 wide, with 2 conspicuous cuspules. Coxae of palp (maxillae) 1.95 long, 0.95 wide, with about 10 conspicuous cuspules ventrally. Sternum 3.35 long, 2.85 wide, with irregularly shaped sigilla in the centre (Fig. 2B).

Legs light brown, light-colored ventrally, with long and short brown sparse setae. All tarsi with tadpole-shaped trichobothrial hairs besides the normal ones. Basal part of tibia III with a saddle-like depression dorsally (Fig. 2C). Tibia and tarsus of palp, distal three segments of legs I and II with bands of short thorn-like spines laterally; tibia III with 4 short spines distally (Fig. 2C). Femur III thickest. Scopulae and claw tufts absent. Palpal claw with a single branched tooth; legs each with 3 tarsal claws, paired claws with two denticles. Leg formula: 4132. Measurements: palp 6.90 (2.70 + 1.10 + 1.60 + 1.50), leg I 8.00 (3.00 + 1.40 + 1.85 + 1.00 + 0.75), leg II 7.55 (2.50 + 1.60 + 1.60 + 0.85 + 1.00), leg III 7.95 (2.75 + 1.50 + 1.45 + 1.00 + 1.25), leg IV 10.30 (3.15 + 1.80 + 1.90 + 1.90 + 1.55).

Opisthosoma black, scattered with thick and slender black setae. Spinnerets brownish, PMS one-segmented, 0.50 long, PMS-PMS 0.20; PLS three-segmented, 0.60 long, thicker and shorter (Fig. 2D). Genitalia with a pair of spermathecae, each stalk slender, long, broader towards the base, distally gradually sclerotized and incurved around 110°, terminating with face-to-face bowl-shaped lobes (Fig. 2E).

#### Distribution.

Guangxi Province (Chongzuo), China.

### Conothele sidiechongensis

Taxon classificationAnimaliaAraneaeCtenizidae

Xu, Xu & Liu sp. n.

http://zoobank.org/D872AF4D-CD97-487B-8DF6-A5DAE371887B

Fig. 3

#### Holotype.

Female (C-YN-005), Sidiechong, Mojiang County, Yunnan Province, China, 23.420°N, 101.676°E, 5 August 2013, collected by D. Li, F.X. Liu and X. Xu.

#### Paratypes.

One female (C-YN-003) collected at Baka Village, Menglun Town, Mengla City, Xishuangbanna, Yunnan Province, China, 21.968°N, 101.210°E, 13 July 2013, collected by F.X. Liu and X. Xu. One female (C-Laos-001), Oudomxay Province, Laos PDR, 27 July 2013, collected by D. Li, F.X. Liu and X. Xu. No male found.

#### Etymology.

‘Sidiechong’ refers to the type locality of the holotype specimen of this species.

#### Diagnosis.

**Figure 3.**
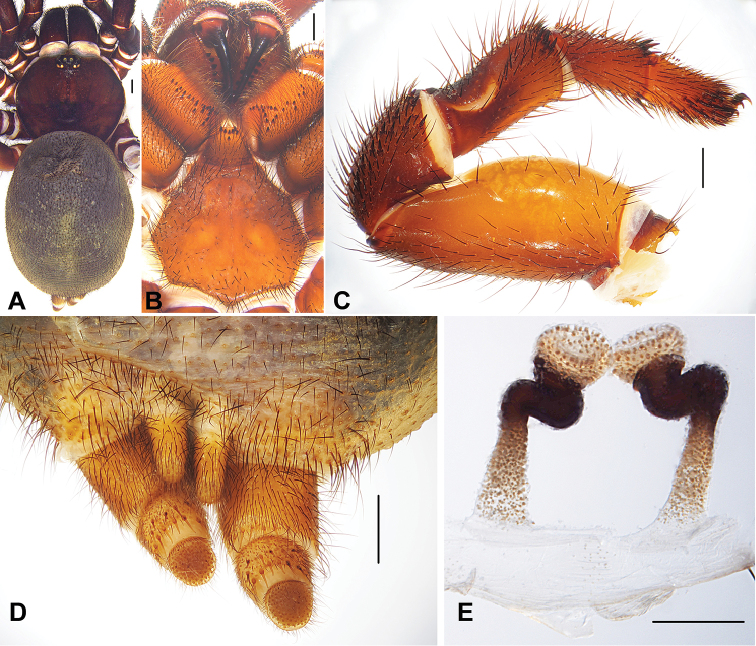
**A** General somatic morphology of *Conothele sidiechongensis* Xu, Xu & Liu, sp. n. (female, C-YN-005) **B** chelicerae, labium, coxae of palp and sternum, ventral view **C** left leg III, prolateral view **D** spinnerets, ventral view **E** female genitalia, dorsal view. Scale bars: **A–D** 1 mm, **E** 0.5 mm.

Female genitalia of *Conothele sidiechongensis* sp. n. resembles to *Conothele taiwanensis* (Tso, Haupt & Zhu, 2003), but can be distinguished from the latter by more or less upwards oriented bowl-shape lobes and stalk bent in zigzag pattern distally (Fig. 3E).

#### Description.

Total length, including chelicerae 25.50; carapace 9.00 long, 9.30 wide; opisthosoma 13.50 long, 10.80 wide. Carapace black brown, black on its margin; with 4 slender setae on eye tubercle and 5 on its back. Caput arched. Fovea deep, strongly procurved and U-shaped (Fig. 3A). Eye tubercle black and low. Eight eyes in two rows, with the anterior eye row slightly procurved and posterior row straight from above (Fig. 3A); eye group 0.60 long, 1.10 wide; ALE-AME 0.25, AME-AME 0.20, PLE-PME 0.05, PME-PME 0.85; MOA 0.60 long, front width 0.86, back width 1.55; ALE: AME: PLE: PME (0.65: 0.33: 0.45: 0.35). Clypeus width 0.45. Chelicerae black, inner margin with 11 teeth, outer margin with 8 teeth. Labium, coxae of palp (maxillae) and sternum black brown (Fig. 3B). Labium 1.60 long, 1.90 wide, with 7 conspicuous cuspules. Coxae of palp (maxillae) 3.60 long, 1.75 wide, with about 29 conspicuous cuspules ventrally. Sternum 5.25 long, 5.90 wide, with large, irregularly shaped sigilla in the centre (Fig. 3B).

Legs black brown, light-coloured ventrally, with long and short brown sparse setae. All tarsi with tadpole-shaped trichobothrial hairs besides the normal ones. Tibia III with a saddle-like depression dorsally (Fig. 3C). Tibia and tarsus of palp, distal three segments of legs I and II with bands of short thorn-like spines laterally; tibia III with 4 short thorn-like spines distally (Fig. 3C); femur III thickest. Scopulae and claw tufts absent. Palpal claw with a single branched tooth; legs each with 3 tarsal claws, paired claws with two denticles, one big and one small. Leg formula: 4123. Measurements: palp 14.88 (5.00 + 2.53 + 3.75 + 3.60), leg I 16.85 (6.00 + 3.00 + 4.00 + 2.10 + 1.75), leg II 16.05 (5.80 + 2.50 + 3.75 + 2.15 + 1.85), leg III 15.80 (4.90 + 3.50 + 3.10 + 2.10 + 2.20), leg IV 19.90 (6.10 + 3.50 + 4.10 + 4.10 + 2.10).

Opisthosoma black, scattered with thick and slender black setae. Spinnerets brownish, PMS one-segmented, 1.20 long, PMS-PMS 0.10; PLS three-segmented, 2.50 long, thicker, covered with brown spines, apical segment dome-shape (Fig. 3D). Genitalia with a pair of spermathecae, with bowl-shaped lobes facing up at the terminal part, long stalks, slightly broader at the basal part, strongly sclerotized and bent in zigzag pattern at the distal part (Fig. 3E).

#### Distribution.

Yunnan Province (Mojiang, Mengla), China; Oudomxay Province, Laos.

### Conothele yundingensis

Taxon classificationAnimaliaAraneaeCtenizidae

Xu, Xu & Li sp. n.

http://zoobank.org/3D374070-85D6-4177-82E1-A575F883D74D

Fig. 4

#### Holotype.

Female (XUC-2014-001+), Mt. Yunding, Tengchong City, Yunnan Province, China, 25.805°N, 98.800°E, 16 December 2014, collected by D. Li, F.X. Liu and X. Xu. No male found.

#### Etymology.

‘Yunding’ refers to the type locality of this species.

#### Diagnosis.

**Figure 4.**
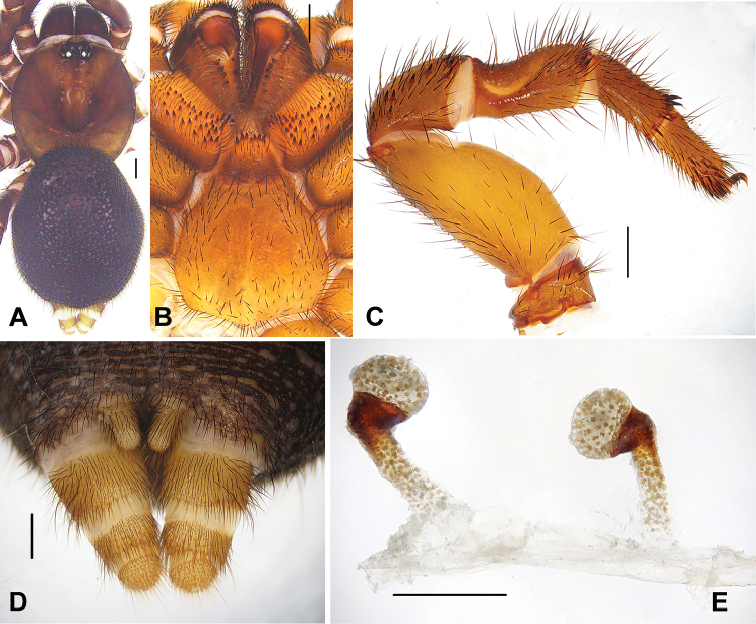
**A** General somatic morphology of *Conothele yundingensis* Xu, Xu & Li, sp. n. (female, XUC-2014-001+) **B** chelicerae, labium, coxae of palp and sternum, ventral view **C** left leg III, prolateral view **D** spinnerets, ventral view **E** female genitalia, ventral view. Scale bars: **A–D** 1 mm, **E** 0.5 mm.

Female of *Conothele yundingensis* sp. n. can be distinguished from *Conothele daxinensis* sp. n. by the slightly upwards and globular lobes terminally (Fig. 4E), can be distinguished from *Conothele baiyunensis* sp. n. by the distal part of stalks bent towards inside about 90° (Fig. 4E).

#### Description.

Total length, including chelicerae 18.30; carapace 7.00 long, 6.80 wide; opisthosoma 9.10 long, 7.90 wide. Carapace black brown, black on its margin; with 9 slender setae on eye tubercle and 3 on its back (Fig. 4A). Caput arched. Fovea deep, strongly procurved and U-shaped (Fig. 4A). Eye tubercle black and low. Eight eyes in two rows, with the anterior eye row procurved and posterior row straight from above (Fig. 4A); eye group 0.8 long, 1.8 wide; ALE-AME 0.35, AME-AME 0.20, PLE-PME 0.10, PME-PME 0.65; MOA 0.70 long, front width 0.75, back width 1.45; ALE: AME: PLE: PME (0.65: 0.25: 0.30: 0.40). Clypeus width 0.40. Chelicerae black, inner margin with 4 teeth, outer margin with 8 teeth. Labium, coxae of palp (maxillae) and sternum black brown (Fig. 4B). Labium 1.40 long, 1.30 wide, with 7 conspicuous cuspules. Coxae of palp (maxillae) 2.35 long, 1.40 wide, with about 45 conspicuous cuspules ventrally. Sternum 3.95 long, 4.10 wide, with irregularly shaped sigilla in the centre (Fig. 4B).

Legs black brown, light-coloured ventrally, with long and short brown sparse setae. All tarsi with tadpole-shaped trichobothrial hairs besides the normal ones. Tibia III with a saddle-like depression dorsally (Fig. 4C). Tibia and tarsus of palp, distal three segments of legs I and II with bands of short thorn-like spines laterally; metatarsus III with 6 and tibia III with 2 short thorn-like spines distally (Fig. 4C); femur III the thickest. Scopulae and claw tufts absent. Palpal claw with a single branched tooth; legs each with 3 tarsal claws, paired claws with two denticles. Leg formula: 4132. Measurements: palp 11.40 (4.25 + 1.50 + 3.00 + 2.65), leg I 13.00 (4.25 + 2.00 + 3.10 + 1.75 + 1.90), leg II 12.25 (3.75 + 1.75 + 3.00 + 1.85 + 1.90), leg III 12.50 (4.00 + 2.50 + 2.50 + 1.75 + 1.75), leg IV 13.50 (4.50 + 2.50 + 2.90 + 2.10 + 1.50).

Opisthosoma black, scattered with thick and slender black setae. Spinnerets brownish, PMS one-segmented, 0.40 long, PMS-PMS 0.15; PLS three-segmented, 2.00 long, thicker, covered with brown spines, apical segment dome-shape (Fig. 4D). Genitalia with a pair of spermathecae with slightly upwards oriented globular lobes terminally, each stalk slender, sclerotized and bent towards inside about 90° distally (Fig. 4E).

#### Distribution.

Yunnan Province (Mt. Yunding, Tengchong), China.

## Supplementary Material

XML Treatment for Conothele

XML Treatment for Conothele baiyunensis

XML Treatment for Conothele daxinensis

XML Treatment for Conothele sidiechongensis

XML Treatment for Conothele yundingensis
